# Longitudinal assessment of HIV-1-specific T-cell responses generated during acute subtype C HIV-1 infection and associations with viral set point

**DOI:** 10.1186/1742-4690-9-S2-P272

**Published:** 2012-09-13

**Authors:** M Radebe, Z Ndhlovu, M Mokgoro, L Maphumulo, M Jaggernath, F Chonco, T Ndung'u, BD Walker

**Affiliations:** 1 University of Kwazulu-Natal, Durban, South Africa; 2 Ragon Institute of MGH, MIT and Harvard, Boston, MA, USA

## Background

HIV-1-specific CD8+ T-cell responses generated during acute infection play a key role in determining the course of disease. However, these early responses may disappear as the infection progresses. Tracking the phenotype, functional ability and fate of these early responses may help elucidate features of CD8+ T-cells that contribute to viral control.

## Methods

We characterized the magnitude and breadth of HIV-1-specific CD8+T-cell responses using the overnight IFN-γ ELISPOT assay in 20 HIV-1 subtype C acutely infected, antiretroviral naive individuals as early as 28 days post initial exposure and up to 12 months post-infection. Cultured IFN-γ ELISPOT assays were used to assess early epitope-specific CD8+ T-cell responses that were below the limit of detection by overnight ELISPOT assays at 12 months post-infection.

## Results

The initial CD8+ T-cell responses detected during the decline in acute phase peak viremia were narrowly directed: T-cell responses were directed against an average of three (range 0-6) of the 410 peptides tested. At 12 months post-infection, immune responses had broadened with an average of seven peptides targeted (range 2-11). The majority of persistent T-cell responses targeted epitopes within Gag and Pol (50% and 30%, respectively). The breadth of persistent CD8+T-cell responses correlated negatively with viral set point (P=0.02). An average of 65% of earlier CD8+ T-cell responses which where not detected by the overnight ELISPOT at later time points, were detected by the cultured ELISPOT assay. There was no correlation between the magnitude or breadth of responses measured using the cultured ELISPOT assay and viral set point.

## Conclusion

These data suggest that persistent CD8+ T-cell responses generated during the earliest stages of HIV-1C infection may play a role in viral control. Furthermore, effector T-cell responses that disappear following acute infection are maintained as memory T-cell responses and our preliminary data suggest that they have no significant impact on viral control.

